# Primary Sinonasal Malignant Melanoma: Effect of Clinical and Histopathologic Prognostic Factors on Survival

**DOI:** 10.4274/balkanmedj.2016.0503

**Published:** 2017-05-15

**Authors:** Sercan Göde, Göksel Turhal, Ceyda Tarhan, Banu Yaman, Gülşen Kandiloğlu, Kerem Öztürk, İsa Kaya, Raşit Midilli, Bülent Karcı

**Affiliations:** 1 Department of Otolaryngology, Ege University School of Medicine, İzmir, Turkey; 2 Department of Patology, Ege University School of Medicine, İzmir, Turkey

**Keywords:** malignant melanoma, nasal cavity, paranasal sinuses, C-kit, sinonasal malignancy, mucosal melanoma

## Abstract

**Background:**

Mucosal melanoma is a rare malignancy arising from melanocytes of the mucosal surfaces. The pattern and frequency of oncogenic mutations and histopathological biomarkers have a role on distinct tumour behaviour and survival.

**Aims:**

To assess the rate of C-KIT positivity and its effect on survival of surgically treated sinonasal malignant melanoma patients with other histopathological biomarkers and clinical features.

**Study Design:**

Retrospective cross-sectional study.

**Methods:**

Seventeen sinonasal malignant melanoma patients with a mean age of 65.41 (39-86) years were included. Overall survival and disease-specific survival rates were calculated. The impact of age, gender, stage and extent of the disease, type of surgery, and adjuvant therapies were also taken into consideration. The effect of mitotic index, pigmentation, S100, HMB-45, Melan-A and C-KIT on survival were evaluated.

**Results:**

Median tumour size was 20 mm (interquartile range=27.5 mm). Pigmentation was present in 7 (41.2%) cases. Median number of mitoses per millimetre squared was 11 (interquartile range=13). Melan A was positive in 7 (41.2%) patients, ulceration was present in 6 cases (35.3%), and necrosis was present in (47.1%) 8 cases. Six patients (35.3%) were positive for S100, 14 (82.4%) specimens stained positive for HMB-45 and C-KIT (CD117) was positive in 9 cases (52.9%). Three patients (16.7%) developed distant metastasis. Five year overall and disease free survival rates were 61.4% and 43.8%, respectively.

**Conclusion:**

Although C-KIT positive sinonasal malignant melanoma patients (52.9%) can be candidates for targeted tumour therapies, the studied clinical or histopathological features along with C-KIT seem to have no significant effect on survival in a small group of patients with sinonasal malignant melanoma.

Mucosal melanoma is a rare, life-threatening malignancy arising from melanocytes of the mucosal surfaces. Unlike cutaneous melanomas, mucosal melanomas constitute approximately 1% of the melanoma cases diagnosed in the United States (1). The incidence of primary sinonasal malignant melanomas (SMM) varies between 0.3 and 2% of all malignant melanomas (2). Though mucosal melanoma can originate from any mucosal site, the sinonasal region is the most common (59-80%) location in the head and neck region (3,4). Melanoma of the sinonasal tract most commonly occurs in the elderly, with a mean presentation age of 64.3 years (5).

The nasal cavity is the predominant location, accounting for approximately 80% of the melanomas arising from the sinonasal tract (2). The remaining 20% of the melanomas originate from the paranasal sinuses. While septum and the lateral wall are the most common sites of origin within the nasal cavity, maxillary and ethmoid sinuses are the most commonly involved sinuses (6,7).

The diagnosis of SMM is usually challenging due to subtle complaints and is therefore diagnosed at an advanced stage. Primary SMM has a more aggressive oncological behaviour and a poorer prognosis than other subtypes of melanomas. Five-year survival rates range from 22% to 80% with median survival times as low as 19 months (2,7,8).

Surgery with or without adjuvant radiotherapy is the treatment of choice in SMM. Due to the rarity of the disease, there is no evidence-based guideline for the staging and treatment of SMM. Subtypes of melanomas show different degrees of oncogenic mutations such as BRAF and C-KIT (9,10). The pattern and frequency of oncogenic mutations have a role on the distinct tumour behaviour and survival. Also, there has been a lot of effort in this field on targeted therapies (9,10).

The aim of this study was to assess the rate of C-KIT positivity and its effect on survival of surgically treated SMM patients along with other histopathological biomarkers and clinical features.

## MATERIALS AND METHODS

This study was carried out at the otolaryngology and pathology departments of a tertiary medical centre. All procedures performed in studies involving human participants were in accordance with the ethical standards of the institutional and/or national research committee and with the 1964 Helsinki declaration and its later amendments or comparable ethical standards. The study was approved by the institutional review board (IRB approval number: 16-8156). The medical records of nineteen patients who underwent surgery for SMM between 2003 and 2014 were included in the study. For this type of study, formal consent is not required.

### Patient selection

Nineteen patients who underwent surgery for mucosal sinonasal melanoma were included in the study. All of the patients had a biopsy-proven malignant melanoma of the sinonasal tract by an experienced pathologist with standard morphological and staining characteristics. Two patients were excluded from the study because sinonasal tissue was involved secondarily by conjunctival malignant melanoma. Eventually, 17 primary SMM patients were included. Patients with distant metastasis were excluded.

### Procedure and instrumentation

Patients were evaluated and decision for surgery was made after a full otolaryngological examination and detailed nasal endoscopy. In order to assess the extent of disease and search for distant metastases, computed tomography, magnetic resonance (MR) or positron emission tomography scans were obtained before surgery. Endoscopic resection or combined technique was performed related to the extent of the disease. One patient required craniofacial resection, two required lateral rhinotomy and 14 were managed endoscopically. Tumour-free margins were obtained by frozen sections during surgery. Nodal disease was managed with neck dissection. All patients received adjuvant radiotherapy. Adjuvant chemotherapy and biotherapy were given based on the general health of the patient.

**Figure 1:**
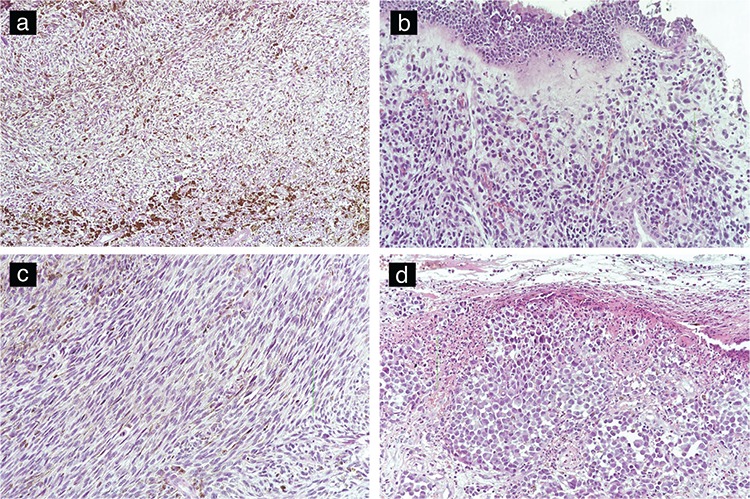
Pigmented spindle cell melanoma (H&E, 100x magnification) (a), cluster of epithelioid mucosal malignant melanoma under the mucosa (H&E 200x magnification) (b), focal melanin pigment is demonstrated on the left upper corner in a spindle cell tumour (H&E 200x magnification (c) and mucosal malignant melanoma with ulceration (H&E 200x magnification) (d).

**Figure 2:**
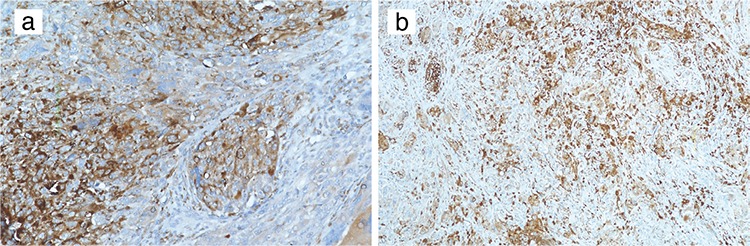
The images at 200x magnification (a) and x100 magnification (b) show immunohistochemical staining for C-KIT (CD117).

All of the tumour specimens were examined in the pathology department. Tumour size was measured during macroscopic pathologic examination. H&E stained slides belonging to all blocks of the cases were re-evaluated. Tumour cells were classified as ovoid, small cell, epithelioid, pleomorphic and spindle-cell (Figure 1). The presence of melanin pigment, necrosis and ulceration was noted. Mitotic index was found by calculating the number of mitoses per millimetre squared. All of the cases were analysed for the following immunohistochemical (IHC) markers: S100, HMB-45, Melan A and C-KIT. C-KIT is a receptor tyrosine kinase protein also known as CD117 and has a role in cell survival, proliferation and differentiation (11) (Figure 2). The pathological findings were correlated with survival outcomes.

The biotin-free, HRP multimer-based, hydrogen peroxide substrate and 3,3’-diaminobenzidine tetrahydrochloride (DAB) chromogen containing ultraView^TM^ Universal DAB Detection Kit (Catalogue number 760-500, Ventana Medical Systems, Tucson, AZ) and a full automated immunohistochemistry staining device (Ventana BenchMark XT, Ventana Medical Systems, Tucson, AZ) were used as the IHC staining system.

Tissue sections were taken on electrostatically charged slides (X-traTM, Surgipath Medical Industries, Richmond, Illinois, USA) and dried at 60 °C for at least two hours. The whole IHC staining process including deparaffinisation and antigen-revealing procedures was performed at the BenchMark XT and fully automated IHC staining device.

Only the primary antibodies S100, HMB-45, Melan A, and C-KIT were manually dropped and incubated at 37 °C for 32 minutes. The specimens were then counterstained with haematoxylin for 8 minutes and post-counterstained with bluing reagent for four minutes. The sections were made transparent with xylene and covered manually. Cytoplasmic positivity of at least 20% for S100, HMB-45, Melan A, and C-KIT was considered positive.

Tumour stage was classified according to the tumor-node-metastasis (TNM) classification for cancer staging designated by the American Joint Committee on Cancer (AJCC) (12). Two different TNM stages were established for each patient: one according to the AJCC tumour staging for mucosal melanoma (*T), and one according to the AJCC tumour staging for nasal cavity and paranasal sinuses (**T).

Follow-up controls were made every three months in the first two years, every six months between the third and fifth year and annually thereafter. Patients had a full otolaryngological examination with special care given to the endoscopic examination. MR imaging of the maxillofacial region and neck lymphatics were obtained in every follow-up visit. Mean follow-up time was 59.76 months (range 10-138). Patient records were retrospectively reviewed and demographic data, clinical information, treatment modalities and outcomes of the patients were evaluated. Survival data were obtained both from patient records and national death index for the correctness of the information and determination of the cause of death.

### Outcome measures

Overall survival and disease-specific survival rates were calculated to estimate survival function. The impact of age, gender, stage and extent of the disease, type of surgery, and adjuvant therapies were also taken into consideration. Besides, the effect of a variety of pathological and IHC parameters such as mitotic index, pigmentation, S100, HMB-45, Melan-A and C-KIT on survival were evaluated. Prognostic factors which have been included in the statistical analysis are shown in Table 1.

### Statistical analysis

Statistical analysis was performed using computer software (SPSS version 22.0, SPSS Inc. Chicago, IL, USA). Chi-square (χ) exact test was used for the comparison of categorical data while independent and paired samples t tests were used for the analysis of parametric variables, Wilcoxon and Mann-Whitney U tests were used for the analysis of non-parametric variables based on the distribution pattern of the data. The distribution pattern was determined by Shapiro-Wilk test. Correlation analysis was performed via Spearman or Pearson correlation analysis depending on the type of variable. Receiver operating characteristic analysis was applied for scale variables which might be a factor for selected criteria. Survival analysis was made with Kaplan-Meier and the effects of multiple independent factors were evaluated with Cox regression analysis. Cox regression analyses were performed separately for the clinical features, and pathologic characteristics. Data were expressed as “mean (standard deviation)”, percent (%), minimum-maximum, odds ratio; 95% confidence interval (CI) and “median [interquartile range (IQR)]” where appropriate. P<0.05 was considered as statistically significant. All p values are derived from univariate analysis except Cox regression test.

## RESULTS

Nine (52.9%) of the patients were female and 8 (47.1%) were male with a mean age of 65.41 (range 39-86) years.

### Clinical features

Tumours originated from the nasal septum in 7 (41.2%) patients, lateral nasal wall and ethmoid sinuses in 7 (41.2%) patients and maxillary sinus in three (17.6%) patients (Table 2). Invasion of the orbit was present in two patients (11.2%) and invasion of the skull base in one patient (5.9%). Eight patients (47.1%) had *T3, 7 (41.2%) had *T4a and two had *T4b (11.8%) disease. Ten patients had (58.8%) **T1, two had (11.8%) **T2, two had (11.8%) **T3 and three had (17.6%) **T4 tumours. Regional lymph node metastasis was present in 4 patients (23.6%). Age, gender, regional metastases, *T and **T stages had no impact on mortality and recurrence (p>0.05). During follow-up three patients (16.7%) developed distant metastasis.

### Histopathological characteristics

Median tumour size was 20 mm (IQR=27.5 mm). Pigmentation was present in 7 (41.2%) cases. Median number of mitoses per millimetre square (mm^2^) was 11 (IQR=13). Melan A was positive in 7 (41.2%) patients. While ulceration was present in 6 cases (35.3%), necrosis was present in (47.1%) 8 cases. Six patients (35.3%) were positive for S100. While 14 (82.4%) specimens were stained positive for HMB-45, C-KIT (CD117) was found positive in 9 (52.9%) (Table 3). C-KIT, necrosis, mitoses number, Melan A, HMB-45, S100 or tumour size had no statistically significant impact on mortality or recurrences (p>0.05).

### Survival

**Figure 3:**
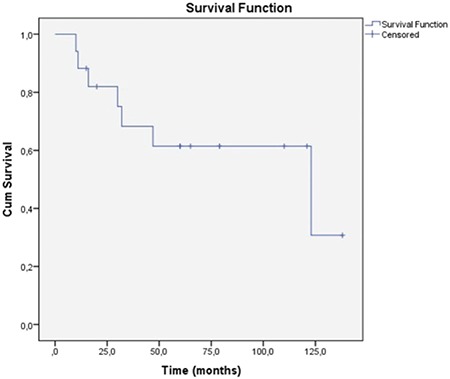
Five year overall survival.

Five year overall and five year disease free survival rates were found to be 61.4% and 43.8% respectively (Figure 3). Median time interval for recurrence was 44.5 months (3-132 months). Prognostic indicators regarding demographic, pathological and IHC data were analysed.

Cox regression analysis of clinical features showed that the origin of the tumour, regional metastases, tumour size, *T or **T stage had no statistically significant impact on either overall or disease free survival rates (p>0.05).

Cox regression analysis of the pathologic characteristics revealed that the number of mitoses, staining positive for Melan A, S100, HMB-45, presence of necrosis or C-KIT positivity had no statistically significant effect on survival function (p>0.05).

## DISCUSSION

Mucosal melanoma of the sinonasal tract is a rare tumour and most of the medical literature relies on case series from single institution reports. Generally, SMM is more common in the nasal cavity than paranasal sinuses. SMM is typically seen in the 6th to 8th decades with no significant sex predilection (2,12,13). Mean age was 65.41 (39-86) years with a slight female predominance (Female:Male=9:8). Gender did not influence the outcomes in the study.

Like all sinonasal malignancies SMM also presents with unspecific symptoms like nasal blockage, epistaxis and facial pressure. Therefore, most of the patients are diagnosed at an advanced stage, particularly if the tumour originates from the paranasal sinuses. Various staging systems had been proposed for the staging of SMM (14,15,16,17). The use of different staging systems made it difficult to obtain meaningful prognostic data from the previous studies. The TNM stage was established according to the seventh edition of AJCC in the study (18). Even mucosal disease is staged as T3 in this staging system addressing the aggressive behaviour of SMM. Patients were also staged according to the AJCC tumour staging for nasal cavity and paranasal sinuses. None of the above-mentioned staging systems had a significant value in the prediction of disease outcome. Houtte et al. (19) compared the prognostic value of two tumour staging classifications in SMM and reported that paranasal sinus involvement was a major prognostic factor. The predictive value of staging systems should be evaluated in multi-centre studies with larger patient numbers.

The origin of the tumour has been reported to have association with prognosis. Roth et al. (20) and Dauer et al. (8) reported that tumours originating in the ethmoid and maxillary sinuses have the worst prognosis because of the late onset of symptoms and close localization to the skull base and orbit. Tumour origin had no impact on SMM patient survival in this study. A statistically significant effect of invasion of the orbit and skull base on SMM outcomes could not be achieved due to the small sample size.

Treatment of SMM relies on complete surgical excision with tumour free margins. The addition of radiotherapy is still controversial. SMM is considered a radioresistant tumour; however, adjuvant radiotherapy may show some benefit by increasing cancer-specific survival (21). There is no established criteria to treat the patients with postoperative radiotherapy and/or chemotherapy. Thus the decision to apply radiotherapy and chemotherapy is made by the treating physician. In a recent study, it was suggested that implementing adjuvant radiotherapy might not provide a survival benefit to SMM patients as survival was poor regardless of adjuvant radiation status (22). Additionally, Samstein et al. (23) reported no survival benefit but improved local control in SMM patients receiving radiotherapy. Endoscopic or combined open surgery was applied in all patients. All of the patients who were treated with surgery received postoperative radiotherapy. Adjuvant radiotherapy is an institutional policy for SMM patients. The choice of implementing adjuvant chemotherapy or biotherapy such as Interferon 2 alpha was based on the general health status of the patient and decided by the medical oncologist.

IHC and pathologic features of SMM is similar to that of cutaneous melanoma. These markers are S100 protein, HMB-45, melan A, microphthalmia transcriptase factor, tyrosinase, vimentin and cytokeratine. Amelanocytic tumours arising from the sinonasal cavity may be mistaken for other sinonasal tumours such as sinonasal undifferentiated carcinoma, lymphoma and olfactory neuroblastoma (24,25). While Roth et al. (20) and Thompson et al. (25) reported that 59% and 67% of the tumours had melanin pigment, Mochel et al. (26) found that 68% of the tumours had no or little melanin. Pigmentation was present in 7 (41.2%) cases in our study.

Several pathological and IHC features were examined including median tumour size, pigmentation, mitotic index, ulceration, necrosis, Melan A, S100, HMB-45 and C-KIT. In a recent study, the presence of >2 mitoses/mm^2^ and necrosis was correlated with tumour progression and overall survival. They included all patients with SMM; however, in this study, only surgically treated patients were included. Such a difference may be attributed to the different patient selection criteria.

Despite radical surgery and adjuvant radiotherapy and/or chemotheapy SMM usually carries a poor prognosis with reported 5 year survival rates ranging from 22% to 80% (2,5,8). In a multicentre study, follow-up of 155 patients revealed a 5-year overall survival rate of 40.1% (27). In the current study 5 year overall and 5 year disease free survival rates were found to be 61.4% and 43.8% respectively. Ten patients (58.8%) had locoregional recurrence. The presence of recurrence had no statistically significant impact on mortality (p>0.05). Cox regression analysis of the demographic, pathological and IHC data revealed that none of the parameters had an impact on survival.

**Figure 4:**
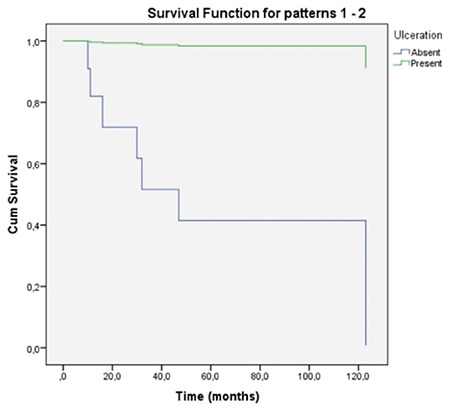
Survival according to C-KIT positivity.

There is a lot of ongoing research regarding immunological and targeted therapies. Administration of interleukin-2 and interferon α as adjuvant immunotherapies was shown to have no improvement on life expectancy (28). C-KIT is a protein encoded by the proto-oncogene KIT, mutations of which may play a role in the early stages of tumour development (29). Imatinib is a monoclonal antibody which targets C-KIT proteins in tumours carrying C-KIT mutations. Clinical response was recorded in patients receiving C-KIT inhibitors in previous studies (30). However, the expression of C-KIT shows a considerable variance between different reports. Liu et al. (31) reported a C-KIT positivity of 85.7% among 28 Chinese patients whereas Zebary et al. (32) reported a C-KIT frequency of only 4%. They also assessed BRAF and NRAS mutations which are associated with targeted therapies and their frequency was also low, like C-KIT (4% and 14% respectively). Cutaneous and mucosal melanomas share common mutations; however, the frequency of BRAF mutation is significantly higher arising in the trunk and skin without chronic sun damage compared to mucosal melanomas (31). Mucosal melanomas more commonly harbour mutations of C-KIT compared to <2% in cutaneous melanomas without chronic sun damage (33,34,35). This study is the first to assess the impact of C-KIT on survival function of primary SMM among many other clinical and histopathological features with a long term follow-up (Figure 4). In this study, C-KIT had no impact on survival function in primary SMM, unlike previous studies which reported a significant correlation in mucosal melanomas. Primary SMM might have a different IHC profile from mucosal melanomas of the other sites. This topic should be elucidated in larger series.

There are some limitations and strong points of this study. The small number of patients could be considered a limitation for the power of the study. However, regarding the low incidence of disease and long follow-up period, the IHC outcomes could still be considered of high importance. Adversely, given the few patients with clinical features such as metastasis and the extent of the primary disease, the prognostic power of the clinical features is limited.

**Figure 5:**
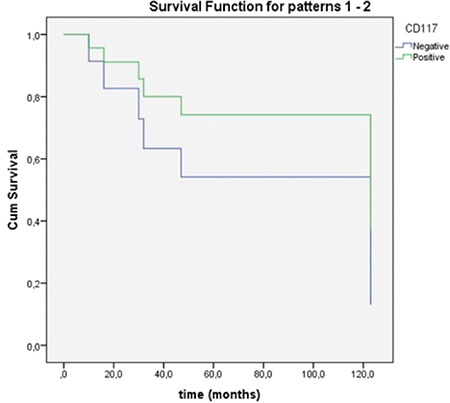
Multiple skip lesions are clearly seen on the resection material (Arrows indicate skip lesions).

Furthermore, surgery for SMM has some points to consider. Skip mucosal lesions may be present in SMM patients and it is advised that both of the nasal cavities should be thoroughly evaluated during surgery with adequate illumination and endoscopic vision. Skip lesions were present in two cases (Figure 5). Skip mucosal lesions had not been clearly defined previously. In particular, this entity may be of great importance during the surgery of small mucosal tumours. Even in the case of open surgery for the removal of large tumours, it is suggested to examine all of the mucosal surfaces in detail with an endoscope.

There some limitations and strong points of this study. Small number of patients could be considered as a limitation for the power of the study. However regarding the low incidence of the disease and long follow-up period, the immunohistochemical outcomes could still be considered of high importance. Adversely, given the few patients with clinical features such as metastasis and extent of the primary disease, limits the prognostic power of the clinical features.

In conclusion, primary sinonasal mucosal melanoma is a rare subtype of malignant melanoma which has an aggressive course with a 5-year survival rate of 61.4%. C-KIT was positive in 52.9% of the SMM’s. C-KIT positive SMM patients can be candidates for targeted tumour therapies. C-KIT positivity was shown to have no effect on survival. None of the clinical or pathological features had a statistically significant prognostic value. The presence of skip mucosal lesions should be considered during surgery to achieve complete removal.

## Figures and Tables

**Table 1:**
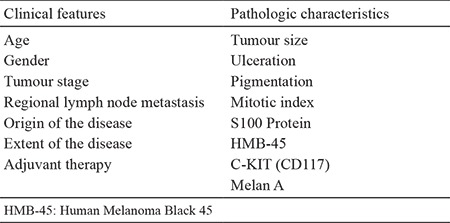
Clinical and pathological variables used as outcome measures in the follow-up of sinonasal malignant melanoma (HMB-45)

**Table 2:**
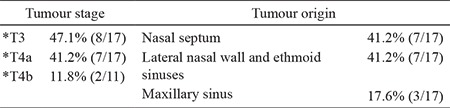
Table demonstrating the tumour stage and origin

**Table 3:**
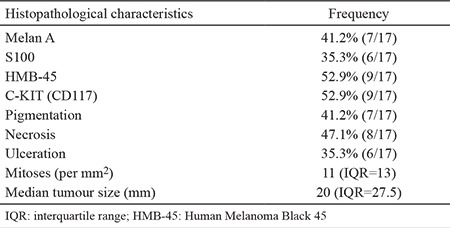
Histopathological characteristics of the sinonasal malignant melanomas patients (HMB-45)
